# Supplementary material for: Are carbon emissions trading and green financial instruments synergistic? -Comprehensive quantitative research based on content analysis
Source: PLoS One. 2024 Mar 7;19(3):e0298601. doi: 10.1371/journal.pone.0298601 (PMC10919601; doi:10.1371/journal.pone.0298601)
Supplement: S1 Data — (ZIP) [file pone.0298601.s001.zip › Data/地级市Z_F工作报告.zip!/╣π╢1⁄2/╔ε█┌╩╨/└┤╫╘╣1⁄2╓┌║┼í╛┬φ┐╦╩2╛▌═°í┐_2012.docx]

各位代表：

　　现在，我代表深圳市人民政府向大会作政府工作报告，请予审议，并请各位政协委员和其他列席人员提出意见。

　　一、2011年工作回顾

　　2011年是深圳经济特区新30年的第一年，是“十二五”起步之年，是深圳“大运”之年。面对复杂多变的外部环境，面对举办大运会的繁重任务，在党中央国务院、省委省政府和市委的坚强领导下，我们贯彻落实科学发展观，加快转变经济发展方式，积极创造深圳质量，完成了五届人大二次会议确定的主要目标任务，实现了“十二五”良好开局。

　　初步预计，本市生产总值突破1.1万亿元，增长10%以上。全口径财政收入超过4000亿元，其中地方财政一般预算收入1339亿元，增长21%。固定资产投资2140亿元，增长10%。社会消费品零售总额3528亿元，增长18%。出口总额2455亿美元，增长20.3%，实现十九连冠。在经济总量高位突破的同时，经济社会发展质量全面提升。

　　——自主创新实现“四个领先”。全社会研发投入占GDP比重3.66%，居全国前列；PCT国际专利申请全球五强企业我市占据两席，企业创新水平全国领先；新一代移动通信、超材料、基因测序和基因组分析等技术全球领先；高新区单位面积产值、税收均居全国首位。基础创新能力建设加速，新增国家级创新载体数量接近此前的总和。获2011年度中国十大创新城市第一名。

　　——结构优化实现“七个高于”。生物、互联网、新能源三大战略性新兴产业整体增速高于GDP增速2倍以上；高新技术产品产值增速高于工业增速7个百分点；现代服务业税收增速高于一般服务业税收增速11.7个百分点；社会消费品零售总额增速高于GDP增速8个百分点；民间投资增速高于固定资产投资增速23个百分点；服务贸易增速高于出口总额增速11个百分点；地方一般预算收入中税收比重近九成，高于全国大城市平均水平。

　　——资源能源消耗实现“三个下降”。万元GDP建设用地下降11.9%；万元GDP能耗和水耗分别下降到0.47吨标准煤和18.7立方米，均为全国最低；化学需氧量、氨氮、二氧化硫、氮氧化物排放量继续下降，超额完成控制目标。用更少的资源消耗和更低的环境代价实现了更有质量的增长。

　　——居民收入和保障水平实现“三个提高”。居民人均可支配收入从3.24万元提高到3.63万元，增长12%；最低工资标准从每月1100元提高到1320元，增长20%；最低生活保障标准从每月450元提高到510元，增长13.3%。

　　——文化建设实现“三个加快”。文化影响力加快提升，深圳“十大观念”影响广泛，“迎风飘扬的旗”等原创精品唱响全国；公共文化服务体系加快完善，市群众艺术馆及六区文化馆均成为国家一级馆，公共图书馆人均图书量居国内大城市前列，公益性文化活动超过1万次；文化创意产业加快发展，增长20.5%，占GDP比重8%，华强文化科技等十家文化领军企业平均增速超过30%。

　　——城市发展实现“三个跃升”。城市基础设施实现跃升，广深高铁、深圳北站、机场二跑道等建成启用，地铁运营里程由22公里增加到178公里，日均客运量超过180万人次；城市面貌实现跃升，全面开展市容环境提升行动，完成1608个城中村综合整治项目、15000多栋建筑刷新改造和46项城市照明提升工程，涌现出深圳湾体育中心、大运中心等一批新地标；人居环境质量实现跃升，新增绿道809公里，深圳湾公园等建成开放，公园总数达到789个，新增日污水处理能力123.5万吨，福田河、新洲河等干流水质明显改善。

　　一年来，我们立足打基础、谋长远，既抓好当年工作，又前瞻布局“十二五”及更长远发展；突出抓创新、调结构，促进产业转型升级；坚持优布局、强功能，提升城市发展质量；着力惠民生、促和谐，加强社会建设，在推动科学发展、创造深圳质量的道路上迈出坚实步伐。

　　（一）举全市之力成功举办大运会，创造了不一样的精彩。办好第26届世界大学生夏季运动会，是中央赋予深圳的光荣使命，是深圳对世界的郑重承诺。胡锦涛总书记亲自出席大运会开幕式并宣布开幕，给我们以巨大鼓舞和激励。党中央国务院和省委省政府的坚强领导，为大运会成功举办提供了重要保证。深圳人民不辱使命，燃烧激情，挥洒汗水，团结拼搏，积极探索简约、创新、绿色、低碳的办会新路，奉献了一届青春活力的体育盛会和一场欢乐祥和的人文盛会，152个国家和地区的12000多名运动员、教练员和随队官员参加大运会，赛会规模和项目数量创历届之最，实现了“从这里开始，不一样的精彩”，谱写了大运会历史上新的辉煌篇章。这是全市人民的骄傲，这是深圳经济特区的荣耀！回顾大运筹办举办的1500多个日日夜夜，不仅唤起我们对大运奋斗历程的美好回忆，而且带给我们富有启示的理性思考。在大运会举办过程中，我们突出创新创意、绿色低碳，强调开闭幕式回归体育、回归仪式，首创火炬虚拟传递，推广使用新能源汽车，广泛采用新技术新材料，有效减少资源能源消耗，在创新大型赛事举办方式上进行了有益探索。我们突出价值提升，办赛事与办城市、惠民生相结合，推动城市建设全面提速，提升人民群众生活品质，广泛开展国际交流合作，充分展示了深圳改革开放创新之城、青春时尚绿色之都的独特魅力。我们突出简约简朴，场馆设施能改不建、能修不换、能租不买、能借不租，实现了赛时保障、后续运营和城市长远发展需求的有机结合。我们突出全民参与，深入开展迎大运创文明活动，127万名志愿者服务大运，818座志愿者服务站遍布全市，市民自愿停驶机动车43万辆，展示了可贵的公民责任和良好的文明素质。历史选择了深圳，深圳无愧于历史。大运会的成功举办，为深圳经济特区创造了弥足珍贵的物质财富、制度财富和精神财富。大运会虽然闭幕，但大运精神将永远激励我们在科学发展道路上开拓创新，奋勇前进！

　　（二）以“十二五”规划纲要为统领，谋划新时期发展战略布局。一是围绕主题主线，立足经济特区发展新定位、新使命，以深圳质量为核心理念，组织编制了“十二五”规划纲要，明确了未来五年发展的指导思想、目标任务、战略举措。二是加快制定82项专项规划，构建了以“十二五”规划纲要为主体，以专项规划、各区规划和功能区规划为支撑的规划体系。三是制定“十二五”规划纲要实施方案，对108项主要目标任务、85项指标、109项重点工程，明确责任主体和进度要求，建立动态分类考核机制，确保规划顺利实施，使“十二五”宏伟蓝图早日成为现实。

　　（三）着眼于构建可持续的竞争优势，提升自主创新能力。一是重大科技基础设施建设取得突破，有力地提升了深圳在国家创新体系中的战略地位。国家超级计算深圳中心投入运行，运算速度全球第四；国家基因库挂牌成立，将为开发利用基因信息资源奠定基础。二是加快建设创新载体，基础创新能力显著增强。新增国家、省、市级重点实验室、工程实验室、工程研究中心和企业技术中心134家，其中国家级20家。三是全方位支持处于科技前沿的新型研究机构加快发展，突破核心技术、关键技术和共性技术。华大基因分别与国际水稻所、美国加州大学戴维斯分校开展国际创新合作，光启研究院在超材料领域一年内申请国内专利和PCT国际专利超过1000件。四是完善自主创新支撑服务体系。加大创新投入，市财政科技投入增长30.6%。大力发展创业投资，新注册超材料、新能源国家创业投资基金等各类股权投资、风险投资基金951家，超过“十一五”注册数总和；创投机构数量和管理资本规模超过全国1/3。中小板、创业板新上市企业分别占全国新增总量的1/10和1/8。落实高层次人才政策，启动人才安居工程，实施“孔雀计划”，引进10个海外高层次创新团队。探索建设专业化、国际化特色学院，华大基因学院挂牌成立。成功举办第十三届高交会和第十届国际人才交流大会。

　　（四）坚持战略性新兴产业和现代服务业双轮驱动，加快新一轮产业转型升级。一是超前布局战略性新兴产业。出台新材料、文化创意和新一代信息技术产业规划及政策，与生物、互联网、新能源形成六大战略性新兴产业规划政策体系。启动建设12个战略性新兴产业基地和11个集聚区，促进新兴产业集聚发展。整合技术、资本、产业等要素资源，组建基因工程、超材料、移动互联网、云计算和新材料等产学研资联盟，推进新兴产业协同创新。坚持以应用促发展，组织实施示范工程，新一代移动通信TD-LTE规模试验启动，基因诊断和新能源汽车等开始规模化应用。二是大力发展现代服务业。积极开展国家服务业综合改革试点。金融业资产规模、效益指标、本外币存贷款余额居全国前列，银行业利润增长26.1%，跨境人民币业务增长2.8倍。证券业资产、利润指标全国第一。深交所新上市公司243家，IPO家数连续两年全球第一。中国资本市场学院启动建设。保险业机构数量和资产总量居全国前列。引进法人金融机构8家。物流业向供应链管理方向发展，顺丰、怡亚通等领军物流企业迅速壮大，深圳港集装箱吞吐量保持全球第四。“文化＋旅游”、“文化＋科技”等文化产业新业态特色凸显，华谊兄弟文化城落户深圳，欢乐海岸建成开业，华侨城集团进入全球旅游景区八强。软件业务收入2250亿元，占全国的1/8。三是推动传统产业转型升级。深圳黄金珠宝业产值超过全国七成，钟表出口总额占全国55%，服装品牌享誉全国。完成710家加工贸易企业转型升级。工业产品内销比重达到46.5%，提高3个百分点。获全省“转变经济发展方式创新工作奖”一等奖。四是以重大项目带动产业高端化。华星光电8.5代液晶面板、深超光电低温多晶硅液晶面板5代线投产，长安标致雪铁龙汽车合资项目、腾讯深圳云计算数据中心奠基。五是引资引智相结合，产业发展和创新引领并举，着力集聚优质高端资源。引进大型总部企业9家。与大唐集团、百度等大型央企、知名民企签约合作项目311项，意向总投资超过8000亿元；与中科院、北大等22所院校签定战略合作协议，引进29项科技经贸人才合作项目。

　　（五）以保障和改善民生为重点，全面加强社会建设。一是加大就业和社会保障力度。实施积极就业政策，财政投入8亿元加强公共就业服务，城镇登记失业率控制在2.2%。扩大社会保障覆盖面，养老、医疗参保人数分别达到760万、1100万。坚决贯彻落实国家房地产市场调控政策。开工建设7.6万套保障性住房，相当于“十一五”开工总量，提前超额完成任务。住房公积金制度全面推行，归集人数居全国大城市第三。二是建立价格调节专项资金，实施最低生活保障标准与物价上涨挂钩联动机制，300家平价农副产品流动商店投入运营。三是教育、卫生、文化、体育事业全面发展。出台中长期教育改革和发展规划纲要，教育体制改革试点全面展开，获全国教育改革创新特别奖。教育投入力度不断加大，市财政教育支出188亿元，增长26.3%。与香港中文大学合作办学正式签约，南方科技大学筹建工作有序推进。基础教育均衡化水平提升，新增公办基础教育学位2.07万个，学前教育、职业教育和民办教育稳步发展。完善基层医疗服务体系，社康中心就诊量达3230万人次，首诊率提高到38%。香港大学深圳医院试运行，市人民医院外科大楼投入使用，儿童医院住院楼等重大卫生项目建设加快。新增三甲医院5家，5个医学学科成为国家级重点学科。精心组织纪念建党90周年等主题文化活动。成功举办第二届国际钢琴协奏曲大赛。关山月美术馆入选全国九大重点美术馆。城市街区24小时自助图书馆达到160台，年借阅量超过110万册次。获全国文化体制改革先进地区称号。文博会规模和影响进一步扩大。全民健身活动广泛开展，深圳运动员在大运会、城运会等重大比赛中取得历史最好成绩。四是加强社会管理和服务创新。社会组织建设取得新进展，“壹基金”落户深圳，举办首届中国?深圳公益项目交流展示会。志愿者之城建设全面启动。连续第七次获全省双拥模范城称号。建成社区服务中心100家，提高了基层服务能力。完善社会工作体系，社工服务专业化、职业化水平提高。推进积分入户工作，1万多名优秀务工人员通过积分入户落户深圳。加强社会治安综合治理，治安形势持续好转。健全应急管理机制，强化食品药品安全监管，全面推行安全生产“一岗双责”，安全生产形势总体平稳。人口计生、统计、档案、民族、宗教、司法、外事、港澳、对台、侨务、气象、民防、防震减灾等各项工作和老年人、妇女儿童、残疾人事业取得新进步。

　　（六）抓住办大运的历史机遇，大幅提升城市功能和生态环境。一是加快区域协调发展。特区一体化扎实推进，市级政府投资的75.4%投向原特区外，重点加强基础设施、公共服务和社会管理。福田中心城区综合功能增强，罗湖服务型经济加快发展，南山创新型城区建设全面推进，盐田现代化滨海城区特征愈益显著，宝安、龙岗城市化水平快速提升，光明、坪山两大新型功能区正在成为新的增长极，龙华和大鹏功能新区设立，形成了定位明确、各具特色、协调互动的区域发展新格局。二是以城市更新释放发展空间。出台城市更新办法实施细则，探索城市发展单元综合开发模式，推动城市发展由依赖增量土地向存量土地二次开发转变，由单一功能开发向整体功能开发转变。大冲村整体改造等24个城市更新项目开工。三是完善城市基础设施。丹平快速一期等道路竣工通车，深惠、水官等道路改造完成，打通33条断头路，取消梧桐山隧道和马田、水田收费站，结束了我市普通公路收费的历史。新增、更新公交运力3493辆，新增公交专用道168公里。原特区外500米公交站点覆盖率由81%提高至85%。“三网融合”试点工作取得积极成效，实现了广电播控平台与电信传输网络的对接。四是强化城市资源能源保障。岭澳核电三期、国家成品油深圳油库、西气东输二线深圳段、迭福LNG项目建设进展顺利，南方电网深圳抽水蓄能电站开工建设，公明供水调蓄、清林径引水调蓄水源工程有序推进。五是加大节能节水力度。投放使用新能源汽车2288辆，累计达3035辆，实现碳减排2.4万吨，成为全球新能源汽车推广力度和应用规模最大的城市。政府机关带头推行合同能源管理，率先实现对大型公共建筑动态能耗监测全覆盖。全市节能建筑面积达到6088万平方米，建筑节能71.6万吨标准煤。水资源消耗增长低于GDP增速7个百分点，被评为全国节水型城市。六是持续改善生态环境。加强大气、水环境治理，完成80%的电厂机组低氮改造，全面推广国IV汽油；新增258公里污水收集管道，龙岗河、观澜河治理力度加大。通过全国首批生态园林城市专家组评审。

　　（七）深化重点领域改革开放，进一步完善科学发展的体制机制。一是投融资体制改革取得重大突破。按照资源、资产、资本一体化运作的思路，建立市场导向的新型投融资体制，调动金融资本，释放投资潜力。组建特区建设发展集团，拓展市地铁集团和投资控股公司功能，增强国有企业投融资和发展能力，市属国有企业服务全市改革发展大局的作用进一步增强。二是开展土地整备制度改革，加大存量土地开发投资力度，启动首期45平方公里的土地整备，为特区未来发展腾出空间。土地管理制度改革总体方案获国家批准，为我市通过先行先试破解用地约束创造了有利条件。三是实施第四轮财政体制改革，以集中财力办大事和调动区级政府积极性相结合为原则，下沉财权和事权，推动管理和服务重心下移。四是完善市级大部门体制改革，已批准建立科技创新委员会，完成区级政府机构改革，公务员分类改革等各项综合配套改革进展顺利。五是提升对外开放水平，引进外商投资项目2470项，合同利用外资增长30.5%。华为、中兴、比亚迪等企业加快全球布局。六是加强区域合作，拓展发展空间。深港跨界基础设施建设取得积极进展，签署落马洲河套地区共同开发等4个深港合作协议。出台前海深港现代服务业合作区条例及管理办法，法定机构运作模式初步建立。推动建立前海部际联席会议制度并召开第一次会议，形成统筹前海开发开放的国家级协调机制，前海先行先试特殊政策已报国家审批。加快深莞惠一体化，启动区域发展总体规划编制工作，取消深惠、深莞边界8个收费站。成立深汕特别合作区。坚决贯彻落实中央要求，对口援疆各项工作全面展开。扎实推进省“双转移”和“双到”工作，在全省产业转移目标考评中名列前茅，深圳经济特区服务全国全省的作用更加突出。

　　（八）以务实创新效率为导向，着力提高政府工作质量。一是认真执行法律法规和人大各项决议，定期向市人大报告、向市政协通报工作，自觉接受人大的法律监督、工作监督，人民政协的民主监督以及社会各界监督，主动听取公众对公共政策的意见建议。加强建议提案办理工作，办理质量进一步提升。二是全面推行绩效管理，对重点工作进行全过程跟踪督办，行政电子监察系统实现对主要行政事项的实时监督和市区街道三级联网监察，市级机关审批事项提前办结率达99.7%。三是以政府投资项目审批制度改革为突破口，简化办事程序，建立跨部门协同办理机制。企业登记前置审批事项大幅精简。四是创新服务方式，市行政服务大厅推出行政审批电话预约服务和延时服务。企业注册逐步实现即来即办。积极开展服务企业活动，及时帮助企业解决实际困难。“深圳政府在线”在中国政府网站绩效评估中蝉联第一。

　　各位代表，过去一年的成绩来之不易，这是党中央国务院、省委省政府和市委正确领导的结果，是全市上下齐心协力、真抓实干的结果。在此，我代表深圳市人民政府，向全市人民，向奋斗在各条战线的广大干部群众，向给予我们不懈支持的人大代表、政协委员、各民主党派、工商联和社会各界人士，向中央和国家有关部门、中央和各省区市驻深单位，向驻深解放军、武警官兵，向所有关心支持深圳经济特区发展的港澳同胞、台湾同胞、海外侨胞和国际友人表示崇高的敬意和衷心的感谢！

　　回顾一年的工作，我们更加清醒地认识到发展中还存在不少问题和困难：一是经济发展的外部压力和土地、资源、环境等内部约束加大，经济增长的内生动力和中心城市的辐射力有待进一步增强；二是人口迅速增长，需求日益多元化，公共服务供给与需求还不平衡，转型期的社会矛盾日益凸显；三是快速城市化积累的各种问题需要加快解决，城市发展水平与世界先进城市相比还有差距；四是科学发展的体制机制有待完善，改革的系统性、协调性和针对性亟需增强；五是行政服务质量和效能尚需提升，在转变发展方式上仍要进一步解放思想、创新思路、加大力度。对于这些问题和困难，我们将认真采取措施，努力加以解决。

　　二、2012年工作思路和重点

　　2012年，是深圳经济特区创新发展、提升质量的关键年，是攻坚克难、转型升级的奋斗年，是开放合作、外溢发展的加速年。我们要准确把握今年工作的阶段性特征，立足新起点，把握新趋势，建立新机制，增创新优势。

　　当前，世界经济形势总体上十分严峻复杂，经济增长放缓，国际贸易增速回落，国际金融市场动荡，经济复苏的不稳定性不确定性上升。我国经济社会在呈现良好态势的同时，发展中不平衡、不协调、不可持续的问题仍然突出，经济增长下行压力和物价上涨压力并存，转型期各种社会矛盾和问题相互交织，区域竞争更加激烈。面对新情况、新问题和新变化，我们既要增强忧患意识，立足当前，保持经济平稳健康较快增长；更要增强机遇意识，着眼长远，把加快转型升级放在第一位，着力提高发展质量，在转型中发展、在创新中提升、在克难中求进。

　　今年政府工作的总体思路是：全面贯彻胡锦涛总书记重要讲话精神，围绕科学发展主题和加快转变经济发展方式主线，按照中央和省的一系列决策部署，紧紧抓住国家支持东部地区率先发展、珠江三角洲地区改革发展、经济特区创新发展的宝贵机遇，弘扬大运精神，想干、敢干、快干、会干，把创新发展、深圳质量贯穿到经济社会发展的各领域和全过程，更加注重追求质量、改善民生、调整结构、扩大内需、改革创新、外溢发展，努力实现有质量的稳定增长、可持续的全面发展，加快建设现代化国际化先进城市。

　　今年全市经济社会发展的主要目标是：本市生产总值增长10％左右，万元GDP能耗和水耗分别下降4.25%和5%。居民人均可支配收入与经济保持同步增长，城镇居民登记失业率控制在3％以内，居民消费价格指数104%左右。上述目标，综合考虑了国际国内发展形势，体现了中央经济工作会议稳中求进的总基调，保持了与“十二五”规划的有机衔接，是在转型升级中追求更高发展质量的目标，是富有挑战、积极进取的目标，需要全市上下凝心聚力、顽强拼搏才能实现。为此，我们将坚定不移地围绕主题主线，优化发展，在政策导向和资源配置上体现有保有压、有扶有控，实施结构性、差异化政策，从产业、财政、土地和环保等方面，引导资源更多地投向自主创新、转型升级、低碳发展和社会建设等领域。重点做好以下九个方面工作:

　　（一）坚持抓创新，加快建设国家创新型城市

　　一是进一步夯实创新基础。全面落实国家创新型城市总体规划实施方案和科技发展“十二五”规划，以自主创新引领深圳未来发展。进一步加大投入，市财政支持技术创新和吸引人才支出增长20.5%。争取更多的国家重大科技基础设施布局深圳，加快建设国家基因库，发挥国家超级计算深圳中心等重大科技基础设施的支撑和服务作用。继续加强创新载体建设，新增各级重点实验室、工程实验室、工程研究中心和企业技术中心100家以上。组织实施50项重大技术攻关项目，引导科研机构、企业积极承担和参与国家科技重大专项，力争在一些前沿和新兴领域取得突破。

　　二是构建充满活力的创新生态体系。继续强化企业自主创新主体作用，完善覆盖创新全链条的支撑服务体系，推动科技创新与价值创造良性互动。做实做好创投引导基金，支持创业投资、天使投资加快发展，完善信用担保、融资租赁和科技保险，助推创新型企业发展。加强知识产权保护和运用，组织开展创新成果应用推广计划，新增20个以上国家高技术产业化发展项目。主动利用全球创新资源，加快引进跨国公司、国际研究机构在深圳设立研发中心，打造国际化创新中心。深入推进“深港创新圈”建设，实现深港两地研发设施、检测平台等创新资源合作共享。加快高新区转型升级，建设国家自主创新示范区。办好第十四届高交会。

　　三是引进培育更多创新人才。坚持引进和培养并重，深入实施“孔雀计划”，集聚海内外优秀人才，力争引进海外高层次创新团队10个以上，支持各类人才在深创新创业。提高深圳大学和清华、北大、哈工大深圳研究生院人才培养和科研能力，加快建设特色学院，完善职业教育体系，大力培育创新人才和多层次技能人才。继续实施人才安居工程，增加人才公寓供应量，为1万名人才提供实物租售，为10万名人才提供租房货币补贴。

　　（二）坚持调结构，构建以“高新软优”为特征、更具国际竞争力的现代产业体系

　　一是做大做强战略性新兴产业。全面落实六大战略性新兴产业规划及政策，迅速扩大产业规模，增加值占GDP比重提高到27%。推进百度国际总部、海信南方总部等项目落地，加快建设长安标致雪铁龙汽车、比亚迪汽车研发生产基地、阿里巴巴国际运营总部等高端产业项目，华星光电项目实现达产，引进一批具有核心技术创新能力和广阔市场前景的新兴产业项目。力争大沙河创新走廊等战略性新兴产业基地和集聚区在落实用地、提升功能、引进项目上取得实质性进展。

　　二是提升现代服务业发展水平。落实国家服务业综合改革试点方案，加快前海深港现代服务业合作区开发开放，强化经济中心城市的服务辐射功能。推进金融创新，集聚金融资源，打造全国金融要素交易、创业投资、财富管理和中小创新型企业融资中心。加强与香港金融业合作，积极发展跨境人民币业务。促进物流业向国际化、高端化、低碳化发展，重点发展供应链管理、物流总部经济、航运衍生服务等高端物流业态。建设国家服务外包示范城市和中国软件名城。做强会计、审计、法律、咨询等专业服务业。大力发展总部经济，完善扶持政策，引进大型总部企业10家以上。

　　三是加快传统产业转型升级步伐。把握好力度、步骤和节奏，坚持产业升级与转移合作相结合，产业转型与城市更新相结合，淘汰落后产能与引入新兴项目相结合，全面提升传统产业竞争力。新设5亿元产业转型升级专项资金。鼓励服装、钟表、黄金珠宝等优势传统产业向研发设计、品牌营销等高端环节延伸，向总部型经济发展。完成2000家非法人来料加工企业的不停产转型。坚决淘汰高耗能、高污染项目。推动保税区、出口加工区转型升级。加快社区工业园区升级改造，促进新兴产业、高端项目落地，引导社区经济向更高经济形态转变。

　　（三）坚持扩内需，保持经济协调稳定增长

　　一是积极扩大消费。调整收入分配结构，继续提高低收入者收入水平，增加中等收入人口比重，释放消费潜力。拓宽和开发消费领域，培育服务消费。加快建设国家电子商务示范市，大力发展网络购物等新型业态，促进网络经济、实体经济互动发展。引进大型商贸企业和采购中心、营运中心、供应链管理中心，完善核心商圈和社区商业网点布局，加快罗湖国际消费中心建设，营造舒适便利的消费环境。社会消费品零售总额增长16.5%。

　　二是加大投资力度。扩大投资规模，提高投资质量，全年固定资产投资增长12%。推进资源资本化、资本证券化，强化特区建发、地铁和投控三大集团融资功能，创新运营模式，提高可持续发展能力。推动企业发行商业票据和债券，形成投资主体多元化、融资渠道多样化的投融资格局。扩大社会投资，引导民间资本进入新兴产业、公用事业和民生领域，社会投资比重达到75%以上。综合平衡经济产出、资源消耗、环境代价等因素，严把产业准入门槛，引进一批投资强度大、产业带动性强的重大项目。实行重大项目全程服务，明确责任，跟踪落实2011年招商引资、招研引智活动成果。促进重大项目快速落地，形成开工一批、建设一批、竣工一批、储备一批的滚动开发格局。建立市、区、部门分级负责的投资项目定期会审制度，提高审理效率。

　　三是巩固和扩大对外贸易。坚持国际化战略，提升开放型经济水平。主动适应国际市场需求新变化，推出促进外贸出口的一揽子措施，努力扩大出口。坚持出口市场多元化战略，巩固欧美等传统出口市场，大力开拓东盟、南美、中东等新兴市场。支持企业用好世贸组织规则，有效应对贸易摩擦。积极转变外贸发展方式，优化出口结构，扩大具有自主知识产权、自主品牌和高附加值的产品出口，提高一般贸易和服务贸易出口比重。积极扩大进口。

　　四是拓展国内市场。落实国家扩大内需政策，支持企业加强内销产品研发，强化品牌和质量优势，增强深圳产品竞争力。构建跨区域政府合作平台，发挥商会、中介机构作用，选择重点地区设立贸易中心和产品营销机构，举办深圳产品推介活动，组织企业参加内地经贸洽谈会、展销会，进一步拓展国内市场，力争工业产品内销比重提高到50%左右。

　　五是加大企业服务力度。高度重视外部环境给企业带来的不利影响，梳理应对金融危机政策，出台更具针对性的措施，大力扶持企业特别是民营企业和中小微型企业发展。深入开展服务企业活动，建立市、区、部门快速协调机制，形成重大问题定期会商和领导挂点联系企业制度。实施中小企业创新帮扶行动计划，扶持中小企业加快技术改造、提升创新能力、加强品牌建设。加大金融对实体经济的支持力度，积极稳妥发展小额贷款业务，完善中小企业融资担保机制。加强行业协会建设，发挥其服务企业、促进产业发展的功能。

　　（四）坚持惠民生，以更大力度提升人民生活质量

　　一是加大民生投入。牢牢把握保障和改善民生这一根本目的，把尊重民意、吸纳民智、凝聚民心贯穿于为民服务的全过程，建设与经济发展水平相适应的民生幸福城市。突出需求导向，加大民生投入，着力解决重点难点问题。市政府投资计划中，社会建设和民生投入比重达83.6%。市财政安排教育支出增长32%，卫生支出增长35.7%，保障性住房支出增长32%，就业支出增长44%，公共交通支出增长32.8%。创新保障和改善民生的制度性安排，形成全社会关心民生、共建民生的良好局面。

　　二是提高社会保障水平。坚持以创业带动就业，鼓励自主创业、自谋职业和灵活就业。加大就业扶持力度，多渠道开发就业岗位，帮助困难人员就业。促进企业切实履行社会责任，构建和谐劳动关系。继续提高最低工资标准和最低生活保障标准，新增5亿元专项资金，重点用于对困难群体的帮扶。进一步扩大社会保险覆盖面，完善养老、失业、劳务工医疗保险等制度。完善住房公积金制度，健全公共租赁房、安居型商品房和货币补贴相结合的多层次住房保障体系。继续加强保障性住房建设，确保完成年度开工任务，提高竣工率。落实国家房地产市场调控政策，促进房地产市场健康发展。用好价格调节专项资金，继续推出平价商店，努力稳定物价总水平。抓好“菜篮子”工程，加快平湖农产品物流园建设，广开渠道，保障农副产品供应稳定。

　　三是加快发展社会事业。落实中长期教育改革和发展规划纲要，深化教育体制综合改革，提升教育核心竞争力。推动高等教育国际化、跨越式发展，完成南方科技大学一期工程和筹办工作，加快与香港中文大学合作办学。新增公办义务教育学位1.55万个，普高学位6750个，建成市第六高级中学和龙岗高级中学。推动学前教育普惠发展，新办幼儿园70所，新增学位2万个。完善家庭、学校、社会三位一体的德育体系，促进学生全面发展。加快新安医院、新明医院、宝荷医院等医疗卫生项目建设，新增病床数3000张，逐步实现医疗卫生设施均衡布局。提高社康中心医疗服务水平，让市民就近享有较好的医疗服务。引进一批国家级重点医学团队，集聚高层次医疗人才，启动80个医学重点学科建设，提高医疗卫生科研水平和实力。鼓励民营医疗和教育机构发展。大力发展养老事业，构建居家养老和机构养老相结合的社会养老服务体系。积极发展残疾人事业。加强双拥共建工作。坚持经济效益和社会效益有机统一，加强大运场馆等体育设施后续开发利用。办好市第八届运动会和首届体育大会。

　　（五）坚持促和谐，加强社会管理创新

　　一是强化基层基础建设。推进社区织网工程和固本强基工程，进一步理顺居委会、社区工作站、业委会、物业公司关系，创新基层公共服务体系，完善协同治理机制，增强社区自治能力。新增社区服务中心100家，提升社区公共服务水平。大力发展社区服务业，满足居民多样化需求。加强人口管理和计划生育工作，进一步优化人口结构，提高人口素质，促进人口与城市协调发展。

　　二是促进社会组织健康有序发展。培育发展与规范管理并重，增强社会组织自我发展、自我管理能力。制定扶持政策，大力培育和发展经济型、公益型、服务型社会组织。加快转变政府职能，将社会组织能够接得住、管得好的事逐步转移过去。创新社会工作机制，提升社工综合素质。

　　三是建设社会主义法治城市。深入推进法治社会建设，以一流的法治环境增强深圳的竞争优势。坚持依法行政，进一步规范行政执法，强化执法监督，建设法治政府。加强法制宣传教育，增强公民法治意识，营造法治社会人人参与、全民共建的良好氛围。创新虚拟社会管理，推动网络立法，净化网络环境。加快建立失信惩戒和守信激励制度，积极构建社会诚信体系。

　　四是提升社会安全水平。健全综治信访维稳工作机制，预防和化解各类社会矛盾，维护社会和谐稳定。完善社会治安立体防控体系，推进平安深圳建设。强化安全生产“一岗双责”，落实企业安全生产主体责任，全面排查治理安全隐患，加强校车安全管理，防范重特大安全事故的发生。加强食品药品安全监管，总结推广大运会食品药品安全管理经验，加大检验检测力度，提高抽检率，健全追溯体系，强化执法检查，严厉查处违法行为，保障食品药品安全，让市民吃得放心、用得安心。做好民防、三防、气象和防震减灾工作，完善应急预案，提高预防和处置各类突发事件的能力。

　　（六）坚持创文明，持续推进文化强市建设

　　一是建设美好的精神家园。加强社会主义核心价值体系建设，繁荣哲学社会科学，培育城市人文精神，提升市民文明素质。发扬大运精神，开展多种形式的精神文明创建活动，积极推进公民道德建设，形成良好的社会风尚，增强城市凝聚力和市民归属感。发展壮大义工队伍，继续开展公务员志愿行动，完善志愿服务体系，加快建设志愿者之城。深入实施关爱行动，办好首届中国公益慈善项目交流展示会，积极发展慈善事业，彰显深圳的包容感恩情怀。

　　二是完善公共文化服务体系。推进公共文化服务均衡发展，创建全国公共文化建设示范区，打造钢琴之城、图书馆之城和设计之都。加快建设当代艺术馆等重大文化项目和基层文化设施，扩大24小时自助图书馆规模，办好深圳读书月、创意十二月等公益文化活动，让市民享受到更加便捷多样的公共文化服务。深入实施文学、影视、音乐、舞台艺术等创作工程，加大文化人才培育引进力度，不断推出“深圳创造”的文艺精品。加强重点文物、改革开放历史文化和非物质文化遗产的保护，丰富城市文化底蕴。

　　三是争当文化产业发展领头羊。设立文化创意产业专项资金，“十二五”期间财政投入25亿元，支持创意设计、动漫游戏、数字出版等文化创意产业发展。鼓励优势文化企业兼并重组，培育一批有竞争力的文化龙头企业。加快建设一批特色鲜明的文化创意产业基地和集聚区。坚持保护优先、适度开发，将大鹏半岛打造成集文化、旅游、生态为一体的新型功能区。做强文博会、中国文化产业投资基金等国家级平台，为文化产业发展提供有力支撑。

　　（七）坚持提品质，打造宜居宜业的城市环境

　　一是以科学的城市管理和先进的硬件设施，全面提升现代化国际化水平。进一步拓宽国际视野，把国际化理念融入到城市发展的方方面面。加强城市管理，完善长效机制，努力实现管理精细化、服务人性化，在软实力上向国际先进城市看齐。强化综合交通枢纽城市地位，建成新航站楼，加快厦深铁路、深圳东站等重大交通工程进度，推动深中通道、深茂铁路、赣深客运专线规划建设。加快推进轨道交通三期工程。深化公交体制机制改革，建设公交都市，公交机动化出行分担率提高到54.5%。继续打通断头路，改善交通微循环。完善慢行交通系统。加快建设“智慧深圳”，以新一代移动通信、下一代互联网和光纤宽带网络为重点，打造一流的信息基础设施，家庭宽带用户达到350万户，无线宽带网络覆盖率达到85%以上。构建多元化的能源资源保障体系，推进南方电网深圳抽水蓄能电站、LNG接收站等项目，完成西气东输二线工程建设，积极推动西江引水工程，逐步形成双气源、双水源供应格局，保障城市供气供水安全。

　　二是加快特区一体化，促进协调发展。根据新的财政体制，政府财力进一步向基层、向原特区外倾斜，区级财力每年增加150亿元以上。按照组团化、差异化、特色化发展要求，统筹推进光明、坪山、龙华、大鹏功能新区和民治、坂田一体化先行示范区开发建设。加快南坪快速二期、坂银通道、坪盐通道等规划建设，打通特区一体化交通瓶颈。加快完善原特区外公共服务设施，公交500米站点覆盖率提高到87%，每千人病床数增加到2.4张，新增普高学位和95%的新增义务教育学位布局在原特区外，提高基本公共服务一体化水平。

　　三是节约集约利用土地，提高空间质量。综合运用城市更新、土地整备、城市发展单元等手段，实施土地二次开发，加速释放一批优质空间。推进岗厦河园、大冲村、坂田宝吉等城市更新项目，通过土地整备累计释放65平方公里可建设用地，推动航空城、华为科技城、笋岗—清水河片区等一批重点片区的综合开发。推动土地开发由地上空间向地下空间延伸，探索城市立体开发模式，拓展空间资源。做好基本农田建设改造工作。积极稳妥处理农村城市化历史遗留问题。强化土地管理共同责任机制，保持查处违法建筑的高压态势。

　　四是加强生态文明建设，探索低碳发展新路。出台低碳发展中长期规划，建立温室气体排放核算、考核及评价机制。完善碳交易市场，促进低碳技术开发应用，开展低碳发展国际合作，推进深圳国际低碳城建设，在新规则中赢得新优势。加大节能减排投入，安排专项资金16亿元，增长128%。推进结构节能、技术节能、管理节能和重点领域节能，新建政府投资项目全部实行绿色建筑标准，推广使用新能源公交大巴1500辆、纯电动出租车500辆。制定先进交通管理政策，倡导绿色出行。试运行企业排污权交易。加大深圳河、龙岗河、坪山河、观澜河、茅洲河等河流治理力度，新增日污水处理能力59.5万吨，新建250公里以上污水管网。加快老虎坑、上洋污泥处理厂建设。研究推进优质水入户工程。改善大气环境质量，加强环保区域合作，综合治理工业废气和机动车尾气，将PM2.5纳入监测范围。积极推进垃圾分类处理，提高循环利用水平。构建“四带六廊”生态安全体系，完善绿化生态系统，人均公园绿地面积达到16.6平方米，新建和整理连通城市绿道和社区绿道1000公里，绿道网总长达到2100公里。

　　（八）坚持增活力，不断深化改革开放

　　一是加大改革攻坚力度。深化综合配套改革，突出系统设计、开放互动、协同推进，加大重点领域和关键环节改革力度，形成有利于科学发展的体制基础、利益导向、政策体系和强大动力。深化社会组织改革，支持引导社会组织参与社会管理和公共服务。深化医疗卫生体制改革，创新公立医院举办和监管机制，实行管办分开，探索医药分开，健全多层次的医保体系。深化住房保障制度改革，完善多层次住房保障体系。深化公务员分类管理和事业单位改革，完善公务员交流制度，探索建立公职人员社会养老保障制度。推进商事登记制度改革，优化营商环境。启动新一轮土地管理制度改革，创新高度城市化地区的土地资源市场化配置模式。探索户籍制度改革，有序提高户籍人口比重，推进基本公共服务均等化。

　　二是实施外溢发展战略。强化深圳全国经济中心城市的服务能力、辐射能力和带动能力，以外溢发展突破城市空间制约，以区域合作优化要素资源配置。积极探索“总部＋分支、研发＋生产、中心＋网络”等外溢发展模式，鼓励深圳企业在输出产品的同时，输出技术、管理和服务。按照优势互补、错位发展、互利共赢的原则，加快深莞惠一体化，完成深莞惠区域发展总体规划，构建更紧密的合作关系。加快深汕特别合作区建设，打造深圳产业转移最佳承接地。加强虚拟空间开发利用，推广应用物联网、云计算等新技术，降低经济社会发展对实体资源和现实空间的依赖。建设国家海洋经济科学发展示范市，培育壮大海洋产业。完善“双转移”长效机制，圆满完成“双到”任务。继续做好新疆、西藏和贵州等对口支援和帮扶工作。

　　三是深化与港澳台合作。加快建设前海深港现代服务业合作区，积极争取前海先行先试特殊政策获国家批准，加大深港联合投资推介力度，突出抓好重点项目落地，尽快形成项目和单元开发亮点，推动前海开发开放大提速。抓住CEPA补充协议八在广东先行先试的机遇，进一步拓宽深港合作领域，提升合作层次。积极推进广深港客运专线、莲塘/香园围口岸、落马洲河套地区合作开发等跨界项目建设，加强深港机场合作，促进要素更便利流动。以文化创意、旅游休闲、中医药产业等领域为重点，进一步加强深澳合作。以落实ECFA协议为契机，提升对台合作水平。

　　四是提高对外开放层次。抓住国际经济形势变化和产业布局深度调整的机遇，鼓励优势企业通过合资、股权并购等途径，加强国际技术合作和人力资源引进。创新利用外资方式，提高利用外资质量，聚焦新兴产业、先进制造业和现代服务业，重点引进总部研发型、绿色低碳类项目，合同利用外资80亿美元以上。加快实施“走出去”战略，支持本土跨国企业全球布局。借助大运会的国际影响，拓展友好交流城市网络。加快构建全方位、深层次、宽领域的开放合作新格局，扩大深圳国际影响力。

　　（九）坚持转作风，推动政府工作提质提效

　　一是加强学习，提高能力。主动适应科技进步、经济转型和社会发展要求，提高理论素养，掌握新知识新技术，以知识结构的转型升级推动经济社会发展的转型升级。完善学习制度，强化考核评价。坚持学以致用，把学习成果转化为谋划发展的思路、促进工作的措施和解决问题的本领，不断增强推动科学发展的能力。

　　二是深入基层，转变作风。加强调查研究，密切联系群众，健全民意收集反馈机制，畅通联系互动渠道，做到知民情、解民忧、纾民困、暖民心。牢固树立正确的政绩观，以“功成不必在我任期”的态度，多做打基础、利长远、谋民利、造福祉的工作。着力克服形式主义、官僚主义，进一步减少会议、文件，严格控制各类评比达标活动。坚持勤俭办事，坚决反对铺张浪费，推动节约型社会建设。

　　三是完善责任体系，狠抓工作落实。围绕“十二五”规划和重点工作，加强行政责任体系建设，逐级分解任务，明确责任，严格重大事项督查督办制度，将办大运的好经验好做法转化为长效机制，以时限倒逼进度，以目标倒逼责任。继续深化审批制度改革，优化业务流程，减少审批环节，提高审批效率和质量。大力发展电子政务，推进信息系统互联互通、资源共享和政府统计四大工程建设。完善政府绩效管理，强化过程控制，加强考评和督查结果的运用，健全激励约束机制，提高行政执行力。

　　四是主动接受监督，建设廉洁城市。自觉接受人大及其常委会的监督，严格执行各项决议决定，积极支持政协履行政治协商、民主监督、参政议政职能，坚持向人大及其常委会报告工作、向政协通报工作，高质量办好建议提案。认真听取民主党派、工商联、无党派人士意见，支持工会、共青团、妇联等人民团体更好地发挥作用。坚持公众参与、专家论证和政府决策相结合，不断提升科学决策、民主决策水平。推进政务公开，接受社会监督和舆论监督，打造阳光政府。将质量意识、绩效理念融入预算管理全过程，严控一般性财政支出，今年行政经费零增长。全面推进廉洁城市建设，加大源头治腐力度，坚决查处违纪违法案件，严厉惩治腐败行为，创造清正廉洁、公平公正的发展环境。

　　各位代表，做好今年工作，任务艰巨，责任重大。让我们以邓小平理论和“三个代表”重要思想为指导，深入贯彻落实科学发展观，按照胡锦涛总书记对深圳经济特区的一系列重要指示，在党中央国务院、省委省政府和市委的坚强领导下，振奋精神，锐意进取，迎难而上，追求卓越，坚持改革开放创新，努力当好推动科学发展、促进社会和谐的排头兵，把深圳经济特区各项事业继续推向前进，以优异成绩迎接中国共产党第十八次全国代表大会胜利召开！
